# The Relationship between Uterine, Fecal, Bedding, and Airborne Dust Microbiota from Dairy Cows and Their Environment: A Pilot Study

**DOI:** 10.3390/ani9121007

**Published:** 2019-11-21

**Authors:** Thuong T. Nguyen, Ayumi Miyake, Tu T.M. Tran, Takeshi Tsuruta, Naoki Nishino

**Affiliations:** 1 Department of Animal Science, Graduate School of Environmental and Life Science, Okayama University, Okayama 700-8530, Japan; nguyenthisongthuong1983@gmail.com (T.T.N.); tsurutafe@okayama-u.ac.jp (T.T.); 2 Okayama Prefecture Livestock Research Institute, Kume 709-3494, Japan; ayumi_miyake@pref.okayama.lg.jp; 3 Faculty of Agriculture and Food Technology, Tien Giang University, My Tho 860000, Vietnam; tranminhtu610@gmail.com

**Keywords:** cowshed, environment, microbiota, uterus

## Abstract

**Simple Summary:**

After calving, dairy cows face the risk of negative energy balance, inflammation, and immunosuppression, which may result in bacterial infection and disruption of the normal microbiota, thus encouraging the development of metritis and endometritis. This study characterized uterine, fecal, bedding, and airborne dust microbiota from postpartum dairy cows and their environment during summer and winter. The results clarify the importance of microbiota in cowshed environments, i.e., bedding and airborne dust, in understanding the postpartum uterine microbiota of dairy cows.

**Abstract:**

The aim of this study was to characterize uterine, fecal, bedding, and airborne dust microbiota from postpartum dairy cows and their environment. The cows were managed by the free-stall housing system, and samples for microbiota and serum metabolite assessment were collected during summer and winter when the cows were at one and two months postpartum. Uterine microbiota varied between seasons; the five most prevalent taxa were *Enterobacteriaceae, Moraxellaceae, Ruminococcaceae, Staphylococcaceae*, and *Lactobacillaceae* during summer, and *Ruminococcaceae, Lachnospiraceae, Bacteroidaceae, Moraxellaceae*, and *Clostridiaceae* during winter. Although *Actinomycetaceae* and *Mycoplasmataceae* were detected at high abundance in several uterine samples, the relationship between the uterine microbiota and serum metabolite concentrations was unclear. The fecal microbiota was stable regardless of the season, whereas bedding and airborne dust microbiota varied between summer and winter. With regards to uterine, bedding, and airborne dust microbiota, *Enterobacteriaceae, Moraxellaceae, Staphylococcaceae*, and *Lactobacillaceae* were more abundant during summer, and *Ruminococcaceae, Lachnospiraceae, Bacteroidaceae,* and *Clostridiaceae* were more abundant during winter. Canonical analysis of principal coordinates confirmed the relationship between uterine and cowshed microbiota. These results indicated that the uterine microbiota may vary when the microbiota in cowshed environments changes.

## 1. Introduction

The postpartum uterus of dairy cows has been shown to be contaminated with diverse bacterial species, including pathogens associated with uterine disease [1,2]. Although one-third to two-thirds of cows remain healthy, others may develop metritis and endometritis; this reduces their food intake and milk production ability and renders them less likely to become pregnant [3,4]. *Escherichia coli*, *Trueperella pyogenes*, *Fusobacterium necrophorum*, and *Bacteroides* spp. (e.g., *Prevotella melaninogenica*) are representative pathogens determined by isolation from the uteri of cows with postpartum uterine disease using the plate-culture method [1]. The understanding of these pathogens has been expanded by employing culture-independent microbiota analyses; several bacterial families, e.g., *Porphyromonadaceae, Mycoplasmataceae, Bacteroidaceae*, and *Leptotrichiaceae*, have been recently considered as pathogens associated with uterine disease [5,6,7].

Restoration of the cervix diameter and regeneration of the uterine epithelium requires three to four weeks [4,8,9]; hence, even in cows with no uterine disease, diverse bacterial species including anaerobes and facultative anaerobes can be detected after parturition. The bacteria contaminating the uteri of postpartum dairy cows are thus considered to originate from feces and the environment. However, data for microbiota in cows from dairy farm environments are limited, and evidence showing the relationship between uterine, fecal, and cowshed microbiota is lacking. Because both *Trueperella* spp. and *Fusobacterium* spp. can be found in the uteri of virgin heifers and pregnant cows [10,11], the belief that the pregnant uterus is sterile until contamination with the environmental bacteria at calving, and that metritis-causing bacteria gain access to the uterus when cows calve should be reconsidered. Regardless, factors affecting uterine microbiota need to be clarified to help prevent uterine disease, improve fertility, and ensure high milk production from the dairy cows.

In this study, a total of 98 samples of uterine mucus, feces, bedding, and airborne dust collected in a dairy farm were analyzed by high-throughput amplicon sequencing. The cows were managed by the free-stall housing system, and samples for microbiota assessment were collected during summer and winter when the cows were at one and two months postpartum. Blood samples were also collected to determine the serum levels of haptoglobin (Hp) and biochemical components. The objective was to characterize the uterine microbiota of postpartum dairy cows during different seasons, and examine if the uterine microbiota was related with the fecal, bedding, and airborne dust microbiota. 

## 2. Materials and Methods

### 2.1. Sampling

A total of 98 samples were collected from cows at the Okayama Prefecture Livestock Research Institute (Okayama, Japan). The cows were housed in a free stall barn and fed total mixed ration silage, which was formulated to contain 500–600 g/kg of dry matter (DM), 160–180 g/kg DM of crude protein (N × 6.25), and 720–740 g/kg DM of total digestible nutrients. The sampling was performed from 6 June to 22 August and from 17 November to 2 March in 2018; hereafter, the former series is referred to as the sampling during summer and the latter, as the sampling during winter. No cows showed symptoms of dystocia and retained placenta, and no visible pus discharge was detected, by the external inspection of the perineum, around the sampling days.

Uterine mucus, blood, and feces samples were collected from nine cows during summer and from eight cows during winter, with two sampling times each at both one (31.0 ± 2.50 days in milk) and two (55.7 ± 5.12 days in milk) months after calving. Uterine and fecal sampling from cows at one and two months postpartum was conducted occasionally on the same day. Accordingly, samples of bedding and airborne dust were collected six times during summer and nine times during winter. 

Uterine mucus was collected by using a cytobrush (Fujihira Industry Co. Ltd., Tokyo, Japan). The cow was restrained, the tail was held, and the perineum area, especially the vulva, was cleaned by wiping with 70% ethanol. The cytobrush instrument was covered with a sanitary plastic sheath and then inserted into the cervix. Inside the cervix, the plastic sheath was ruptured, and the instrument was further passed through the cervix toward the base of the larger horn, at which point the stainless-steel tube was retracted to expose the cytobrush. Uterine mucus was collected by rotating the cytobrush while in contact with the uterine wall [12]; then, the cytobrush was pulled back into the stainless-steel tube to avoid bacterial contamination from the vagina, vulva, and feces. The cytobrush was cut and placed into an Eppendorf tube and stored.

Fecal samples were collected from the rectum and blood samples were taken from the caudal vein. Airborne dust samples were collected by placing three petri dishes approximately 1.0 m above the ground for five minutes; they were then gathered into a tube using sterile physiological saline. Bedding samples were collected from three separate places in a cowshed. In the free stall system, cows could move and rest freely, and determining their resting place was difficult. Thus, a composite sample prepared from three separate samples was regarded as a representative means of assessing the bedding and airborne dust microbiota at the time of sampling. All the samples were kept on ice during their transportation to the laboratory and stored at −20 °C until further analyses. Procedures and protocols for the animal experiments were approved by the Animal Care and Use Committee (OKU-2016290), Okayama University, Japan.

### 2.2. Blood Analyses

The levels of serum albumin (Alb), urea nitrogen (BUN), total cholesterol (T-Cho), non-esterified fatty acids (NEFA), aspartate aminotransferase (AST), alanine aminotransferase (ALT), calcium (Ca), and phosphate were determined by using the respective commercial kits (FUJIFILM Wako Pure Chemicals Co., Tokyo, Japan). The concentration of serum haptoglobin (Hp) was determined by the quantitative sandwich enzyme-linked immunoassay technique using the bovine Hp ELISA kit (Life Diagnostics, Inc., West Chester, PA, USA). The intra- and inter-assay coefficients of variation for these determinations were <10% and <15%, respectively.

### 2.3. DNA Extraction

For uterine samples, the thawed cytobrush was soaked in 1 mL of sterile physiological saline for 30 min to release the uterine microbiota. Bacterial pellets were obtained by centrifugation at 16,000 *g* for 2 min, and then washed with 500 µL of solution I containing 0.05 M D-glucose, 0.025 M Tris-HCl (pH 8.0), and 0.01 M sodium EDTA (pH 8.0). After further centrifugation at 16,000 *g* for 2 min, the bacterial cells were lysed with 180 µL of lysozyme solution (20 g/L lysozyme, 0.02 M Tris-HCl [pH 8.0], 0.002 M sodium EDTA [pH 8.0], and 1.2 g/L Triton X-100) at 37 °C for 1 h. Airborne dust samples dissolved in sterile physiological saline were processed in the same way. Bacterial DNA was purified by using the DNeasy blood and tissue kit (Qiagen, Germantown, MD, USA), according to the manufacturer’s instructions. In case of the fecal and bedding samples, bacterial DNA was extracted following the procedure for the repeated bead beating plus column method [13] and purified using the mini DNeasy stool kit (Qiagen, Germantown, MD, USA). 

### 2.4. Illumina MiSeq Sequencing

Bacterial DNA was amplified by two-step polymerase chain reaction (PCR) to generate amplicon libraries for next-generation sequencing. The primers targeting the V4 region of 16S ribosomal RNA (rRNA) genes (forward: 5′-ACACTCTTTCCCTACACGACGCTCTTCCGATCTGTGCCAGCMGCCGCGGTAA-3′; reverse: 5′-GTGACTGGAGTTCAGACGTGTGCTCTTCCGATCTGGACTACHVGGGTWTCTAAT-3′; tail sequences are underlined) [14] were used for the first round of PCR, with the following protocol: initial denaturation at 94 °C for 2 min, followed by 35 cycles of denaturation at 94 °C for 30 s, annealing at 50 °C for 30 s, elongation at 72 °C for 30 s, and an elongation step at 72 °C for 5 min. The PCR products were purified by electrophoretic separation on a 2.0% agarose gel using a Fast Gene Gel/PCR Extraction Kit (NIPPON Genetics Co., LTD., Tokyo, Japan). The second round of PCR, with adapter-attached primers, followed the protocol of initial denaturation at 94 °C for 2 min, 10 cycles of denaturation at 94 °C for 30 s, annealing at 59 °C for 30 s, elongation at 72 °C for 30 s, and an elongation step at 72 °C for 5 min. The second-round PCR products were purified in the same way as that in case of the first-round PCR products.

The purified amplicons were pair-end sequenced (2 × 250 bp) on an Illumina MiSeq platform at FASMAC Co., Ltd. (Kanagawa, Japan). Raw sequence data were analyzed using the Quantitative Insights Into Microbial Ecology (QIIME version 1.9.0). The 250-bp reads were truncated at any site receiving an average quality score under 20. Truncated reads that were shorter than 225 bp were discarded. In primer matching, sequences showing overlaps longer than 200 bp were assembled. The final reads obtained after pair-end joining were grouped into operational taxonomic units (OTUs) using a 97% similarity threshold. The sequence data were analyzed and categorized from the phylum to the family level using the default settings of the Ribosomal Database Project classifier. The results of the sequence analysis are available in the DDBJ Sequence Read Archive under project identification number PRJDB8863.

### 2.5. Statistical Analysis

Data for milk yield, blood metabolites, and the relative abundances of the major bacterial families (for families present at >1.0% in at least one sample) were subjected to the non-parametric Mann–Whitney U test to examine the effect of season and months after calving. Bacterial abundance data were also subjected to canonical analysis of principal coordinates (CAP) to define the assignment and clustering that explained variations in the microbiota. Discriminant vectors with a Pearson correlation >0.7 were considered significant. Two-way ANOVA and a t-test were performed using JMP (version 11; SAS Institute, Tokyo, Japan) and CAP was carried out using Primer version 7 with the Permanova+ add-on (Primer-E, Plymouth Marine Laboratory, Plymouth, UK).

## 3. Results

The parity of the cows examined during summer and winter were 2.0 ± 0.7 and 2.3 ± 1.3, respectively. Milk yield of the cows was 35–40 kg, and no differences were seen between two seasons and between one and two months after the calving (Table 1). The level of serum Alb was higher during summer than during winter. Numerical differences were also seen for the levels of serum NEFA (summer > winter), AST (summer < winter), and ALT (summer < winter). Likewise, the level of serum T-Cho was higher at two months than at one month postpartum.

**Figure 1:**
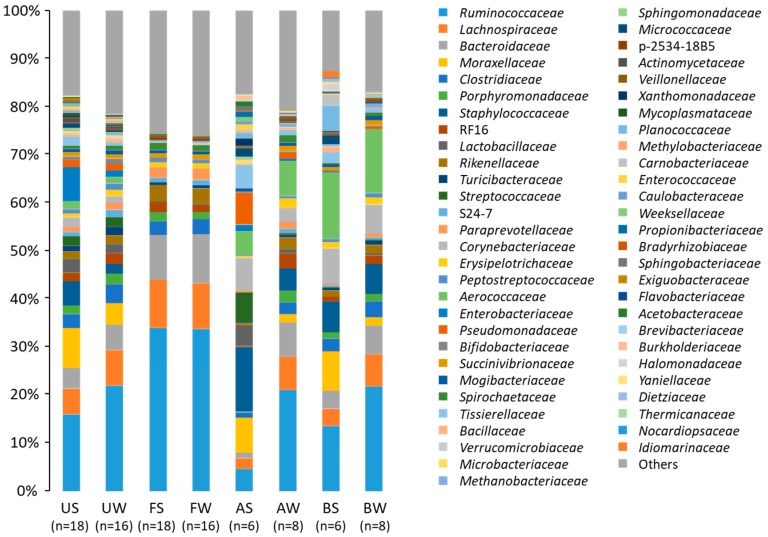
Uterine, fecal, bedding, and airborne dust microbiota in the dairy farm examined during two seasons. AS, AW, BS, BW, FS, FW, US, and UW indicate airborne dust during summer, airborne dust during winter, bedding during summer, bedding during winter, feces during summer, feces during winter, uterine mucus during summer, and uterine mucus during winter, respectively.

The Illumina MiSeq sequencing revealed that the microbiota showed a huge taxonomic diversity, including 34 phyla and 213 families, of which 10 phyla and 41 families were shared among all samples (Figure 1). *Firmicutes, Proteobacteria, and Bacteroidetes* were the three major phyla, and accounted for >90% of the total abundance regardless of the sampling time. The abundance of *Proteobacteria* was higher and those of *Firmicutes* and *Bacteroidetes* were lower during summer than during winter.

Differences owing to sample collection at one and two months postpartum were not found in any families of the uterine microbiota (Table 2). The five most abundant taxa of the uterine microbiota during summer were *Enterobacteriaceae* (12.4%), *Moraxellaceae* (12.1%), *Ruminococcaceae* (11.0%), *Staphylococcaceae* (7.6%), and *Lactobacillaceae* (3.9%), and those during winter were *Ruminococcaceae* (22.3%), *Lachnospiraceae* (7.3%), *Bacteroidaceae* (5.5%), *Moraxellaceae* (4.5%), and *Clostridiaceae* (3.9%). Season-to-season differences were seen for these families; relative abundances of *Enterobacteriaceae, Moraxellaceae*, *Staphylococcaceae,* and *Lactobacillaceae* were greater and those of *Ruminococcaceae, Lachnospiraceae, Bacteroidaceae*, and *Clostridiaceae* were lower during summer than during winter. The abundances of *Actinomycetaceae* and *Mycoplasmataceae* were low, except that the abundance of *Actinomycetaceae* in one cow was 19.2%, and that of *Mycoplasmataceae* in another cow was 11.6% during summer. The relative abundance of *Fusobacteriaceae* was substantially low (0.00–0.43%) in the uterine samples in this study.

The five most prevalent taxa of the fecal microbiota during summer were *Ruminococcaceae* (33.9%), *Bacteroidaceae* (11.3%), *Lachnospiraceae* (9.2%), *Rikenellaceae* (3.2%), and *Clostridiaceae* (3.2%), and those during winter were *Ruminococcaceae* (34.5%), *Lachnospiraceae* (10.1%), *Bacteroidaceae* (9.2%), *Rikenellaceae* (3.4%), and *Clostridiaceae* (3.0%) (Table 3). Differences due to season were not observed for the five most prevalent taxa. The relative abundance of *Mycoplasmataceae* was 0.01% and those of *Actinomycetaceae* and *Fusobacteriaceae* were both <0.005% in the fecal microbiota.

The five most abundant taxa of the bedding microbiota during summer were *Aerococcaceae* (13.8%), *Ruminococcaceae* (10.8%), *Moraxellaceae* (8.3%), *Corynebacteriaceae* (7.3%), and *Staphylococcaceae* (6.6%), and those during winter were *Ruminococcaceae* (17.0%), *Aerococcaceae* (13.3%), *Lachnospiraceae* (6.6%), *Staphylococcaceae* (6.3%), and *Corynebacteriaceae* (6.0%) (Table 4). The relative abundance of *Moraxellaceae* was higher during summer than during winter, and the abundances of *Ruminococcaceae* and *Lachnospiraceae* were lower during summer than during winter. The abundances of *Actinomycetaceae* (0.17%), *Fusobacteriaceae* (0.02%), and *Mycoplasmataceae* (0.02%) were low in the bedding microbiota.

The five most abundant taxa of the airborne dust microbiota during summer were *Staphylococcaceae* (13.4%), *Moraxellaceae* (7.2%), *Corynebacteriaceae* (7.0%), *Pseudomonadaceae* (6.7%), and *Streptococcaceae* (6.1%), and those during winter were *Ruminococcaceae* (17.4%), *Aerococcaceae* (7.3%), *Bacteroidaceae* (7.1%), *Lachnospiraceae* (6.8%), and *Staphylococcaceae* (4.7%) (Table 5). Season-to-season differences were found for the relative abundances of these families, except for *Aerococcaceae*. The abundances of *Actinomycetaceae* (0.20%), *Fusobacteriaceae* (0.12%), and *Mycoplasmataceae* (0.02%) were also low in the airborne dust microbiota.

**Figure 2:**
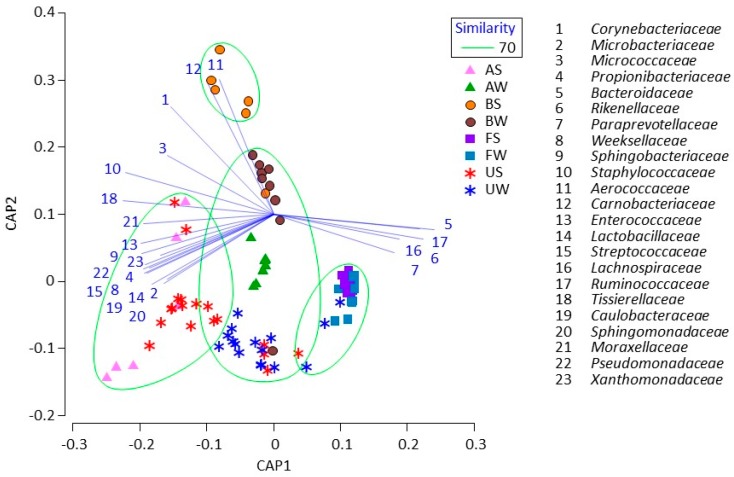
Canonical analysis of principal coordinates plot characterizing the uterine, fecal, bedding, and airborne dust microbiota in the dairy farm. Samples enclosed in a green circle are regarded to be in the same group at a 70% similarity level. Lines indicate discriminant vectors, with a Pearson correlation > 0.7, and the bacterial families in the groupings.

Based on the CAP analysis, during summer, the uterine microbiota was grouped with the airborne dust microbiota, whereas bedding and fecal microbiota formed two other (separate) groups (Figure 2). During winter, most of the uterine microbiota was grouped with the airborne dust and bedding microbiota, whereas some of the uterine microbiota formed the same group with the fecal microbiota.

## 4. Discussion

It has been demonstrated that the level of blood metabolites can help describe postpartum uterine health and the resumption of postpartum cyclicity. Cows with high milk-producing ability may face a severe negative energy balance after calving, and cows with uterine disease may show lower levels of serum Alb, T-Cho, and Ca, and higher levels of serum NEFA, BUN, and AST [15,16,17]. In this study, although the results revealed that the cows showed lower serum Alb and higher serum ALT levels, suggesting a greater risk of uterine disease during winter, they may have had an appropriate protein-energy balance during both seasons, because the level of serum T-Cho was sufficiently high and those of serum BUN and NEFA were acceptably low. The finding that there were no differences between samples collected during different seasons in terms of the serum concentrations of Hp, an acute-phase protein that reflects the severity of inflammatory responses, including metritis [18,19,20], also could support this.

In this study, 24 out of 34 cow samples showed a baseline level (<20 μg/L) of serum Hp. Four out of 10 other samples showed a high Hp level, at >130 μg/L, which was indicated as an upper cut-off value to differentiate between healthy and metritis-affected cows [18]. Regardless, the cows with elevated Hp concentration were indicated to have a normal liver function, i.e., their serum AST and ALT levels were similar to those of others. Likewise, the cows with high Hp levels apparently showed normal uterine microbiota, i.e., low abundances of typical pathogens such as members of *Actinomycetaceae, Fusobacteriaceae*, and *Mycoplasmataceae*. 

In the uterine microbiota examined in this study, *Firmicutes* was the most abundant phylum, followed by *Proteobacteria* and *Bacteroidetes*. Machado et al. [6] and Wang et al. [21] also found that *Firmicutes* was the most abundant phylum, showing abundances of 52.3 and 76.7%, respectively, in the uterine microbiota of healthy cows at around one month postpartum. The predominant taxa of *Firmicutes* in the healthy uterine microbiota, however, is yet to be defined; *Geobacillus* spp. (*Bacillaceae*, Machado et al. [6]), *Lactococcus* spp. (*Streptococcaceae*, Wang et al. [21]), and *Ruminococcaceae* [7] were reported as the most abundant components of healthy uterine microbiota.

Santos et al. [5] and Wang et al. [21] reported that *Proteobacteria* was the most abundant phylum in the uterine microbiota of healthy cows; however, in this study, *Proteobacteria* was the second and third most abundant phylum in the samples obtained during summer and winter, respectively. The predominant taxa of *Proteobacteria* in the healthy uterine microbiota are also unclear; Santos et al. [5] have indicated *Pasteurella* spp., and Bicalho et al. [22] have demonstrated *Escherichia* spp. (*Enterobacteriaceae*), *Shigella* spp. (*Enterobacteriaceae*), and *Pseudomonas* spp. (*Pseudomonadaceae*) as the major taxa of *Proteobacteria.* Our results stated that *Enterobacteriaceae* and *Moraxellaceae* were the two major taxa, which is, in part, similar to the results of the study by Bicalho et al. [22].

The abundance of *Bacteroidetes*, particularly with regards to the families *Bacteroidaceae* and *Porphyromonadaceae,* has been shown to increase in cows with metritis [6,22]. Meanwhile, the standard prevalence of *Bacteroidetes* among the healthy uterine microbiota is yet to be defined. Machado et al. [6] found an increase in the abundance of *Bacteroidetes* from 12.7% in cows without metritis to 18.9% in those with metritis, whereas Bicalho et al. [22] reported an increase in the abundance of *Bacteroidetes* from 20% in healthy cows to 29% in cows showing purulent vaginal discharge. The relative abundance of *Bacteroidetes* in samples obtained during winter (about 20%) in this study could be regarded as both a healthy- and metritis-level abundance. Greater abundance of *Bacteroidetes* during winter was probably because of the greater abundance of this phylum in airborne dust and bedding microbiota during this season.

Even in healthy cows, *Fusobacteria* have been found as a prevalent phylum at one week, and have shown >5% abundance at one month after calving [5,6]. In this study, in all uterine samples, *Fusobacteria* showed a relative abundance of <0.5%, and thus, the data are not shown in Table 2. At two months postpartum during summer, *Moraxellaceae* was found as the most abundant family in the uterine microbiota. Although members of *Acinetobacter*, a genus belonging to the family *Moraxellaceae*, have been reported to be associated with sub-clinical endometritis [21] and repeat breeder [23], *Acinetobacter* spp. can be found in diverse environments and their pathogenicity is regarded to be low. Similar to the finding for *Bacteroidetes*, differences in the prevalence of *Moraxellaceae* at different sampling times could be due to the different abundances of this family in the bedding and airborne dust microbiota during the two seasons.

The average abundance of *Actinomycetaceae* was 2.3% in cows at two months postpartum during summer, because one cow showed an extremely high abundance (19.4%) of this family compared with the other cows (<0.4%). Likewise, the same cow showed a substantially high abundance (3.1%) of *Actinomycetaceae* at one month postpartum. Even in cows with metritis, the prevalence of *T. pyogenes* in the uterine microbiota was as high as 5% at one month postpartum [6,22,24]. The cow with a high abundance of *Actinomycetaceae* may have had uterine infection; however, the levels of serum Hp and other metabolites were normal in this cow.

One cow showed a high abundance (11.6%) of *Mycoplasmataceae* at one month postpartum during summer, and thus, the average abundance value of this family became 1.3%. Although members of *Mycoplasmataceae,* particularly *Ureaplasma* spp., have been considered as metritis-associated pathogens [6,7], a greater abundance (13%) of this family has been detected in healthy cows [7] than was detected in our study, and serum metabolite levels in these cows were normal.

Although researchers have recognized the importance of cowshed microbiota, studies reporting the microbiota in airborne dust on dairy farms are limited. Dutkiewicz et al. [25] examined cowshed microbiota by plate culture and isolated several species, including those belonging to the families *Micrococcaceae, Staphylococcaceae, Bacillaceae, Corynebacteriaceae, Microbacteriaceae, Streptomycetaceae, Moraxellaceae, Enterobacteriaceae,* and *Pseudomonadaceae*. However, *Bacillaceae, Microbacteriaceae*, and *Streptomycetaceae* were not detected at >1.0% in this study. Likewise, Tsapko et al. [26] found that the genera *Staphylococcus* (*Staphylococcaceae*), *Streptococcus* (*Streptococcaceae*), *Escherichia* (*Enterobacteriaceae*), *Klebsiella* (*Enterobacteriaceae*), *Pseudomonas* (*Pseudomonadaceae*), *Citrobacter* (*Enterobacteriaceae*), and *Acinetobacter* (*Moraxellaceae*) were increased in the spring/summer period, which was agreement with our results showing that the relative abundances of these families were greater during the summer than during the winter. However, their studies were aimed to evaluate the potentially harmful effects on the health of exposed workers; hence, it is difficult to interpret their results in relation to the effects on the health of exposed animals.

The CAP analysis clarified that uterine microbiota was related with fecal, bedding, and airborne dust microbiota, and that their relationships may vary between seasons. According to the conventional statistics (Mann–Whitney U test), the fecal microbiota was shown to be stable regardless of the season, whereas uterine, bedding, and airborne dust microbiota were shown to be different in the two seasons. Thus, the conventional statistics did not suggest a significant association between the uterine and fecal microbiota. Meanwhile, the CAP analysis clearly demonstrated the relationship between uterine, bedding, and airborne dust microbiota. During summer, the dairy farm used fans with a mist of water to cool the bodies of the cows; hence, this enforcing ventilation may have caused a difference in the association between the uterine, bedding, and airborne dust microbiota during the two seasons.

## 5. Conclusions

This study indicated the importance of microbiota in cowshed environments in understanding the postpartum uterine microbiota of dairy cows, which might help account for differences between the bacterial taxa among healthy uterine microbiota in previously published studies. Although airborne dust and bedding microbiota can be associated with uterine microbiota, the abundances of *Actinomycetaceae* (0.07–0.45% for airborne dust and 0.03–0.38% for bedding), *Fusobacteriaceae* (0.00–0.42% for airborne dust and 0.00–0.08% for bedding), and *Mycoplasmataceae* (0.00–0.07% for airborne dust and 0.00–0.06% for bedding) in the cowshed were too low to exert large variations of the uterine microbiota. Further research is necessary to understand the complex interactions within and between uterine and cowshed environment microbiota, and differences between the susceptibilities of cows housed in the same environment to uterine infection.

## Figures and Tables

**Table 1:** Milk yield and blood metabolites concentration of dairy cows examined at one and two months postpartum during the two seasons.

Item	Summer		Winter		Mann-Whitney U Test	
	1M (n = 9)	2M (n = 9)	1M (n = 8)	2M (n = 8)	Season	Month
Milk yield (kg/day)	39.0 ± 5.98	39.2 ± 6.93	34.6 ± 11.4	38.2 ± 9.95	NS	NS
Blood metabolites						
Albumin (g/dL)	3.57 ± 0.22	3.56 ± 0.28	3.29 ± 0.32	3.11 ± 0.28	**	NS
BUN (mg/dL)	13.4 ± 2.16	12.6 ± 2.82	11.9 ± 3.64	11.5 ± 2.30	NS	NS
Cholesterol (mg/dL)	151 ± 23.3	197 ± 25.1	148 ± 26.9	175 ± 32.5	NS	**
NEFA (μEq/L)	0.37 ± 0.37	0.20 ± 0.19	0.14 ± 0.74	0.13 ± 0.59	NS	NS
Calcium (mg/dL)	8.92 ± 0.36	9.06 ± 0.67	9.13 ± 1.44	8.74 ± 1.57	NS	NS
Phosphorus (mg/dL)	6.72 ± 0.75	5.69 ± 1.14	5.84 ± 0.86	5.84 ± 0.93	NS	NS
AST (U/L)	45.1 ± 3.82	52.5 ± 6.60	55.2 ± 11.8	52.2 ± 4.78	NS	NS
ALT (U/L)	11.1 ± 2.55	12.5 ± 1.90	12.7 ± 2.21	13.9 ± 1.22	NS	NS
Haptoglobin (μg/L)	19.4 ± 4.62	101 ± 168	151 ± 276	42.3 ± 65.4	NS	NS

Summer and winter stand for the sampling conducted between 6 June and 22 August and between 17 November and 2 March, respectively. The terms 1 M and 2 M indicate one and two months postpartum respectively. BUN; blood urea nitrogen, NEFA; non-esterified fatty acid, AST; aspartate aminotransferase, ALT; alanine aminotransferase. **: *p* < 0.01, NS: not significant.

**Table 2:** Relative abundance (%) of uterine microbiota of the dairy cows examined at one and two months postpartum during the two seasons.

Phylum/Family	Summer		Winter		Mann-Whitney U Test	
	1M (n = 9)	2M (n = 9)	1M (n = 8)	2M (n = 8)	Season	Month
*Actinobacteria*	7.31 ± 6.78	7.27 ± 6.20	5.04 ± 2.66	3.50 ± 2.92	NS	NS
* Actinomycetaceae*	0.53 ± 0.96	2.34 ± 6.32	0.37 ± 0.22	0.23 ± 0.23	NS	NS
* Bifidobacteriaceae*	0.14 ± 0.16	0.17 ± 0.16	1.30 ± 0.60	1.06 ± 0.79	**	NS
* Corynebacteriaceae*	2.75 ± 2.49	2.03 ± 0.91	1.76 ± 0.97	1.15 ± 1.03	NS	NS
* Micrococcaceae*	1.67 ± 2.09	1.17 ± 0.46	0.38 ± 0.29	0.25 ± 0.24	**	NS
*Bacteroidetes*	14.5 ± 12.9	12.2 ± 8.61	19.1 ± 4.74	22.1 ± 5.28	**	NS
* Bacteroidaceae*	3.57 ± 3.63	3.09 ± 2.14	4.77 ± 1.29	6.18 ± 1.96	**	NS
* Paraprevotellaceae*	0.73 ± 0.77	0.78 ± 0.82	1.40 ± 0.95	1.62 ± 0.78	**	NS
* Porphyromonadaceae*	2.38 ± 3.43	1.16 ± 0.89	2.06 ± 0.51	2.23 ± 0.65	**	NS
* RF16*	1.23 ± 1.42	1.31 ± 1.44	1.91 ± 1.69	2.19 ± 1.00	*	NS
* Rikenellaceae*	1.36 ± 1.38	1.07 ± 0.87	1.65 ± 0.88	2.08 ± 0.70	*	NS
* S24-7*	0.35 ± 0.35	0.28 ± 0.13	2.10 ± 1.83	1.11 ± 0.71	**	NS
*Firmicutes*	44.2 ± 7.65	42.1 ± 4.43	55.8 ± 5.71	59.7 ± 4.70	**	NS
* Aerococcaceae*	2.30 ± 3.26	1.01 ± 0.35	1.22 ± 0.67	1.42 ± 1.31	NS	NS
* Bacillaceae*	0.50 ± 0.60	0.27 ± 0.12	1.28 ± 3.06	0.11 ± 0.12	NS	NS
* Clostridiaceae*	1.78 ± 0.94	1.46 ± 0.54	3.88 ± 2.13	4.01 ± 0.62	**	NS
* Erysipelotrichaceae*	0.50 ± 0.42	0.46 ± 0.19	1.40 ± 0.64	1.29 ± 0.48	**	NS
* Exiguobacteraceae*	0.09 ± 0.10	1.01 ± 1.96	0.14 ± 0.22	0.04 ± 0.05	NS	NS
* Lachnospiraceae*	3.57 ± 2.69	3.12 ± 1.42	6.63 ± 2.06	7.97 ± 1.79	**	NS
* Lactobacillaceae*	3.05 ± 1.98	4.67 ± 1.22	2.82 ± 1.91	1.28 ± 0.85	**	NS
* Peptostreptococcaceae*	0.38 ± 0.22	0.45 ± 0.16	1.26 ± 0.59	1.38 ± 0.89	**	NS
* Ruminococcaceae*	11.8 ± 10.9	10.1 ± 5.84	19.6 ± 6.76	24.9 ± 5.56	**	NS
* Staphylococcaceae*	8.14 ± 5.85	7.12 ± 2.88	2.45 ± 1.17	1.75 ± 1.57	**	NS
* Streptococcaceae*	2.03 ± 1.18	2.44 ± 0.92	1.96 ± 1.42	1.69 ± 1.48	NS	NS
* Tissierellaceae*	2.80 ± 1.73	3.02 ± 1.33	0.83 ± 0.48	0.60 ± 0.62	**	NS
* Turicibacteraceae*	0.33 ± 0.19	0.33 ± 0.13	2.10 ± 1.19	1.56 ± 0.99	**	NS
*Proteobacteria*	29.0 ± 16.0	35.0 ± 10.5	13.5 ± 6.72	8.40 ± 4.42	**	NS
* Enterobacteriaceae*	11.9 ± 9.19	12.8 ± 4.80	1.41 ± 0.84	1.12 ± 1.04	**	NS
* Moraxellaceae*	9.94 ± 6.15	14.2 ± 4.94	5.87 ± 6.14	2.99 ± 2.82	**	NS
* Pseudomonadaceae*	1.51 ± 0.97	2.32 ± 1.06	1.63 ± 0.88	0.83 ± 0.64	*	NS
* Succinivibrionaceae*	1.28 ± 2.33	0.97 ± 0.79	0.71 ± 0.48	1.10 ± 0.89	NS	NS
*Tenericutes*	2.54 ± 3.69	1.13 ± 0.63	2.33 ± 1.64	2.62 ± 0.48	**	NS
* Mycoplasmataceae*	1.32 ± 3.87	0.02 ± 0.02	0.41 ± 1.07	0.10 ± 0.09	*	NS

Phyla and families having a relative abundance of >1% in at least one sample are indicated. Summer and winter stand for the sampling conducted between 6 June and 22 August and between 17 November and 2 March, respectively. The terms 1 M and 2 M indicate one and two months postpartum, respectively. **: *p* < 0.01, *: *p* < 0.05, NS: not significant.

**Table 3:** Relative abundance (%) of fecal microbiota of the dairy cows examined at one and two months postpartum during the two seasons.

Phylum/Family	Summer		Winter		Mann-Whitney U Test	
	1M (n = 9)	2M (n = 9)	1M (n = 8)	2M (n = 8)	Season	Month
*Bacteroidetes*	32.2 ± 4.55	31.6 ± 2.90	28.6 ± 2.27	30.8 ± 5.06	NS	NS
* Bacteroidaceae*	10.3 ± 2.78	12.3 ± 2.92	9.68 ± 2.10	8.65 ± 1.76	NS	NS
* Paraprevotellaceae*	2.55 ± 1.10	2.27 ± 0.88	1.61 ± 1.15	2.70 ± 0.63	NS	NS
* Porphyromonadaceae*	1.47 ± 2.00	1.26 ± 0.69	2.13 ± 0.99	1.85 ± 1.15	*	NS
* RF16*	1.01 ± 0.57	1.33 ± 0.91	1.51 ± 0.49	2.48 ± 1.55	*	NS
* Rikenellaceae*	3.13 ± 0.61	3.20 ± 0.80	3.42 ± 0.40	3.37 ± 0.78	NS	NS
* S24-7*	1.39 ± 0.58	1.46 ± 0.41	0.79 ± 0.52	1.27 ± 0.54	*	NS
*Firmicutes*	60.2 ± 4.38	60.1 ± 2.92	63.1 ± 3.48	60.8 ± 5.42	NS	NS
* Clostridiaceae*	2.89 ± 1.05	2.89 ± 0.68	3.04 ± 0.94	2.87 ± 0.63	NS	NS
* Erysipelotrichaceae*	1.22 ± 0.51	1.44 ± 0.39	0.94 ± 0.20	1.26 ± 0.56	NS	*
* Lachnospiraceae*	9.32 ± 4.64	9.06 ± 2.30	9.38 ± 1.67	10.7 ± 4.56	NS	NS
* Ruminococcaceae*	33.8 ± 5.05	33.9 ± 1.87	37.2 ± 2.46	31.7 ± 3.28	NS	*
*Proteobacteria*	1.35 ± 0.87	1.72 ± 0.85	2.15 ± 1.39	1.32 ± 0.61	NS	NS
* Succinivibrionaceae*	1.11 ± 0.93	1.15 ± 0.84	1.16 ± 1.09	0.72 ± 0.65	NS	NS
*Spirochaetes*	0.94 ± 0.72	1.18 ± 0.60	1.93 ± 1.02	1.72 ± 1.16	*	NS
*Spirochaetaceae*	0.92 ± 0.71	1.12 ± 0.60	1.72 ± 1.01	1.67 ± 1.17	*	NS

Phyla and families having a relative abundance of >1% in at least one sample are indicated. Summer and winter stand for the sampling conducted between 6 June and 22 August and between 17 November and 2 March, respectively. The terms 1 M and 2 M indicate one and two months postpartum, respectively. *: *p* < 0.05, NS: not significant.

**Table 4:** Relative abundance (%) of bedding microbiota of the dairy farm cowshed examined at two seasons.

Phylum/Family	Summer (n = 6)	Winter (n = 9)	Mann-Whitney U Test
*Actinobacteria*	11.4 ± 3.90	6.97 ± 3.60	*
* Corynebacteriaceae*	7.28 ± 2.89	6.01 ± 3.48	NS
* Micrococcaceae*	1.78 ± 0.92	0.27 ± 0.19	**
*Bacteroidetes*	11.4 ± 5.24	16.8 ± 3.80	*
* Bacteroidaceae*	3.75 ± 2.08	5.95 ± 1.61	NS
* Porphyromonadaceae*	1.28 ± 0.40	1.71 ± 0.67	NS
* RF16*	0.93 ± 0.59	1.65 ± 0.67	*
* Rikenellaceae*	1.04 ± 0.55	1.74 ± 0.36	*
*Firmicutes*	60.2 ± 2.48	66.7 ± 3.49	**
* Aerococcaceae*	13.8 ± 2.66	13.3 ± 6.92	NS
* Carnobacteriaceae*	2.21 ± 0.48	0.63 ± 0.31	**
* Clostridiaceae*	2.11 ± 0.25	2.45 ± 0.30	NS
* Erysipelotrichaceae*	1.32 ± 0.31	1.56 ± 0.33	NS
* Lachnospiraceae*	3.40 ± 1.22	6.59 ± 1.76	**
* Mogibacteriaceae*	0.70 ± 0.24	1.01 ± 0.17	*
* Peptostreptococcaceae*	1.18 ± 0.36	1.75 ± 0.45	*
* Planococcaceae*	5.65 ± 4.32	0.09 ± 0.20	**
* Ruminococcaceae*	10.8 ± 5.35	17.0 ± 2.31	*
* Staphylococcaceae*	6.62 ± 2.32	6.27 ± 2.82	NS
* Tissierellaceae*	2.32 ± 1.30	1.11 ± 0.53	*
*Proteobacteria*	13.8 ± 5.22	4.37 ± 1.90	**
* Halomonadaceae*	1.33 ± 0.93	0.12 ± 0.09	**
* Idiomarinaceae*	1.46 ± 2.12	0.01 ± 0.01	**
* Moraxellaceae*	8.32 ± 3.72	1.80 ± 1.15	**
* Succinivibrionaceae*	0.55 ± 0.36	1.01 ± 0.71	NS

Phyla and families having a relative abundance of >1% in at least one sample are indicated. Summer and winter stand for the sampling conducted between 6 June and 22 August and between 17 November and 2 March, respectively. **: *p* < 0.01, *: *p* < 0.05, NS: not significant.

**Table 5:** Relative abundance (%) of air-borne dust microbiota of the dairy farm cowshed examined at two seasons.

Phylum/Family	Summer (n = 6)	Winter (n = 9)	Mann-Whitney U Test
*Actinobacteria*	12.4 ± 4.64	4.31 ± 0.81	**
* Corynebacteriaceae*	6.95 ± 3.52	2.97 ± 0.36	**
* Micrococcaceae*	1.52 ± 0.84	0.35 ± 0.40	**
* Propionibacteriaceae*	1.13 ± 0.50	0.02 ± 0.02	**
*Bacteroidetes*	5.33 ± 1.68	23.1 ± 1.35	**
* Bacteroidaceae*	1.13 ± 0.85	7.05 ± 0.65	**
* Paraprevotellaceae*	0.35 ± 0.17	1.52 ± 0.11	**
* Porphyromonadaceae*	0.35 ± 0.23	2.41 ± 0.15	**
* RF16*	0.21 ± 0.22	3.01 ± 0.33	**
* Rikenellaceae*	0.44 ± 0.25	2.28 ± 0.26	**
*Firmicutes*	56.9 ± 2.17	58.1 ± 1.44	NS
* Aerococcaceae*	4.75 ± 4.34	7.28 ± 0.95	NS
* Clostridiaceae*	0.80 ± 0.51	2.12 ± 0.14	**
* Erysipelotrichaceae*	0.31 ± 0.25	1.94 ± 0.17	**
* Lachnospiraceae*	2.22 ± 1.48	6.79 ± 0.44	**
* Lactobacillaceae*	4.10 ± 4.44	0.97 ± 0.91	*
* Peptostreptococcaceae*	0.69 ± 0.56	1.00 ± 0.15	NS
* Planococcaceae*	1.12 ± 1.05	0.05 ± 0.04	**
* Ruminococcaceae*	3.73 ± 3.02	17.4 ± 1.13	**
* Staphylococcaceae*	13.4 ± 2.05	4.72 ± 1.26	**
* Streptococcaceae*	6.10 ± 2.50	0.31 ± 1.94	**
* Tissierellaceae*	4.98 ± 0.84	1.09 ± 0.14	**
*Proteobacteria*	22.6 ± 7.21	6.75 ± 1.12	**
* Enterobacteriaceae*	1.32 ± 0.66	0.35 ± 0.35	*
* Moraxellaceae*	7.19 ± 1.94	1.78 ± 0.55	**
* Pseudomonadaceae*	6.72 ± 2.68	1.34 ± 0.31	**
* Xanthomonadaceae*	1.57 ± 0.61	0.07 ± 0.03	**
*Spirochaetes*	0.11 ± 0.11	1.57 ± 0.24	**
* Spirochaetaceae*	0.07 ± 0.08	1.49 ± 0.26	**

Phyla and families having a relative abundance of >1% in at least one sample are indicated. Summer and winter stand for the sampling conducted between 6 June and 22 August and between 17 November and 2 March, respectively. **: *p* < 0.01, *: *p* < 0.05, NS: not significant.
